# Plasma Sphingomyelins as Biomarkers for Diabetic Retinal Neurodegeneration: The Maastricht Study

**DOI:** 10.1016/j.xops.2025.100870

**Published:** 2025-06-27

**Authors:** Siddhita A. Jadhav, Birke J. Benedikter, Sara B.A. Mokhtar, Frank C.T. van der Heide, Govindasamy Kumaramanickavel, Marleen M.J. van Greevenbroek, Carroll A.B. Webers, Tos T.J.M. Berendschot

**Affiliations:** 1 Mental Health and Neuroscience Research Institute, Maastricht University, Maastricht, the Netherlands; 2 University Eye Clinic, Maastricht University Medical Center +, Maastricht, the Netherlands; 3 GenVams Trust, Chennai, India; 4 Department of Internal Medicine, Maastricht University Medical Center +, Maastricht, the Netherlands; 5 School for Cardiovascular Diseases, CARIM, Maastricht University, Maastricht, the Netherlands; 6 Inserm U1153, Epidemiology of Ageing and Neurodegenerative Diseases, Université Paris Cité, Paris, France; 7 Rothschild BRAINlab, Adolphe de Rothschild Foundation Hospital, Paris, France

**Keywords:** Diabetic retinal neurodegeneration, Diabetic retinopathy, Sphingomyelin

## Abstract

**Objective:**

Sphingomyelin (SM) may play a role in the early stages of diabetic retinopathy. Early diagnosis of diabetic retinopathy is crucial for preventing the irreversible vision loss associated with this condition. This study aimed to examine the link between SM and key indicators of diabetic retinopathy, namely retinal neurodegeneration, including corneal nerve measures, retinal layer thickness, and mean retinal sensitivity. Understanding these relationships may help identify early biomarkers and therapeutic targets for preventing or slowing the progression of diabetic retinopathy.

**Design:**

We used data from the Maastricht Study, a large population-based observational cohort with oversampling of individuals with type 2 diabetes mellitus (T2DM).

**Subjects:**

In this study, SM levels were examined across 3 study groups: (1) individuals with normal glucose metabolism; (2) prediabetes; and (3) T2DM.

**Methods:**

Fasting plasma SM levels were measured using the nuclear magnetic resonance platform from Nightingale Health.

**Main Outcomes and Measures:**

Linear regression analysis was conducted to assess the link between total plasma SM (determinant) and indicators of retinal neurodegeneration (outcomes), including corneal nerve measures, mean retinal sensitivity, and retinal thickness, while adjusting for potential confounders affecting SM metabolism.

**Results:**

Among the 3598 individuals examined, the average plasma levels of total SM were significantly lower in individuals with T2DM (*P* < 0.001) than those with prediabetes and the control group, even after stratification by lipid-modifying medication usage. In regression analysis, after full adjustment, lower levels of SM were associated with reduced mean retinal sensitivity: β (95% confidence interval) for the right eye (n = 1934), 0.088 (0.012, 0.164) and for the left eye (n = 1925), 0.111 (0.033, 0.189). However, no significant correlations were found with other indicators of retinal neurodegeneration.

**Conclusions:**

Lower levels of plasma SMs were linked to reduced retinal sensitivity in individuals with diabetes, indicating their involvement in early neurodegenerative alterations in the diabetic retina. These findings suggest that SMs could be explored as potential biomarkers for detecting diabetic retinal neurodegeneration at an early stage of diabetes. However, further research is essential to clarify the biological pathways involved and to evaluate the effectiveness of SMs as clinical biomarkers.

**Financial Disclosure(s):**

The author(s) have no proprietary or commercial interest in any materials discussed in this article.

Diabetic retinopathy, a common complication of diabetes mellitus (DM), involves progressive damage to the retinal neurovascular unit.^1^ Increasing evidence indicates that early retinal neurodegeneration plays a crucial role in the pathogenesis and progression of diabetic retinopathy.^2^ Notably, the loss of synaptic proteins stands as a hallmark of retinal neurodegeneration.^2^ Clinical assessment of retinal neurodegeneration involves observing structural alterations by measuring retinal layer thickness and functional alterations by measuring retinal sensitivity. Prior studies have detected retinal neurodegeneration by measuring a reduction in retinal layer thickness and retinal sensitivity in individuals with diabetes compared with controls.^3^^,^^4^ Moreover, recent research has shown corneal neurodegeneration in diabetic individuals without apparent retinopathy^5^ and hence could be an indicator for early detection of diabetic retinal neurodegeneration (DRN).^6^^,^^7^ However, despite notable advances in comprehending neuronal pathology, the molecular intricacies driving the development of DRN remain elusive.

Sphingolipids, known for their roles in cellular signaling and membrane structure,^8^, ^9^, ^10^ have recently gained attention for their implications in neuroretinal health. Alterations in their metabolism have been implicated in various neurodegenerative disorders, including Alzheimer disease, Parkinson disease, depression, schizophrenia, and Niemann–Pick disease.^11^^,^^12^ Several studies,^13^, ^14^, ^15^, ^16^, ^17^, ^18^, ^19^, ^20^, ^21^ both in animal models and humans, have linked sphingolipid metabolites with DM and its complications. Sphingomyelin (SM), a type of sphingolipid, is a major constituent of plasma membranes, which plays a crucial role in various cellular processes.^11^ Sphingomyelin constitutes roughly 2.5% of the total retinal lipids.

Given the extensive evidence linking sphingolipids to neurodegenerative conditions, we posit dysregulation in SM levels to be associated with DRN pathology and anticipate observing alterations in circulating total SM concentrations when comparing individuals with and without DM. However, to date, circulating SM levels in individuals with DM and their link with early retinal neurodegenerative changes have remained to be investigated. Thus, the present study was designed to explore the links between total SM (determinant) levels in plasma and key indicators of retinal neuronal health measures (clinical outcome measures), such as retinal layer(s) thickness, retinal sensitivity, and corneal neurodegeneration in individuals with healthy controls (normal glucose metabolism [NGM]), prediabetes, and individuals with type 2 diabetes mellitus (T2DM).

Understanding the potential interplay between sphingolipids and these clinical indicators could provide valuable insights into the molecular mechanisms underlying the early stages of DRN. This may lead to the identification of novel biomarkers for the early detection of DRN in individuals with T2DM and also opens avenues for the development of therapeutic intervention.

## Methods

### Study Population and Design

We used data from the Maastricht Study, an observational prospective population-based cohort study. The rationale and methodology have been described previously.^22^ In brief, the study focuses on the etiology, pathophysiology, complications, and comorbidities of T2DM and is characterized by an extensive phenotyping approach. Eligible for participation were all individuals aged between 40 and 75 years and living in the southern part of the Netherlands. Participants were recruited through mass media campaigns and from the municipal registries and the regional Diabetes Patient Registry via mailings. Recruitment was stratified according to known T2DM status, with an oversampling of individuals with T2DM, for reasons of efficiency. The present report includes cross-sectional data from the 3807 participants, who were included in the baseline survey between September 2010 and January 2014. The examinations of each participant were performed within a time window of 3 months. The study has been approved by the institutional medical ethical committee (NL31329.068.10) and the Minister of Health, Welfare and Sports of the Netherlands (Permit 131088-105234-PG) and follows the Declaration of Helsinki. All participants gave written informed consent. The present study was reported as per the STROBE statement for observational cohort studies (and assessed with the STROBE checklist).

### Clinical Measurements

#### Glucose Metabolism Status

Glucose metabolism status (GMS) was assessed and classified according to the World Health Organization 2006 criteria as described previously.^22^ Impaired fasting glucose and impaired glucose tolerance were combined into prediabetes.

#### Ophthalmic Measurements

We included data obtained from corneal confocal microscopy (CCM),^5^ OCT, and perimetry.^23^ For imaging the corneal nerves, we utilized CCM (Heidelberg Retina Tomograph III, Rostock cornea module, Heidelberg Engineering), and imaging was performed as described previously.^5^ Corneal confocal microscopy measurements were performed only in the left eye for logistic reasons. We have analyzed the following indices of corneal neurodegeneration: (1) corneal nerve fiber length; (2) corneal nerve fiber density; and (3) corneal nerve branch density. The CCM evaluations were conducted between April 2013 and January 2017. Participants who had been enrolled in the Maastricht Study before April 2013 were invited for a follow-up visit, referred to as the “catch-up visit.” For these individuals, there was a “CCM lag time,” specifically a median time interval of 5.2 years, between the CCM assessments and all other measurements. The thickness of retinal layer(s) was assessed by OCT (Spectralis unit; Heidelberg Engineering), as described previously,^23^ including retinal nerve fiber layer, ganglion cell layer, inner nuclear layer, inner plexiform layer, outer nuclear layer, outer plexiform layer, photoreceptor 1, photoreceptor 2, and total retinal thickness. Mean retinal sensitivity (in the central and perimacular area) was assessed using a Heidelberg Edge Perimeter (Heidelberg Engineering), as described previously.^23^ OCT and perimetry measurements were performed in both eyes: right eye and left eye.

#### Total SM Measurements

Fasted ethylenediamine tetra-acetate plasma samples were stored at −80° C until analysis. Sample storage time varied from 1 to 15 years. Total SM was quantified from plasma samples of individuals using high-throughput proton nuclear magnetic resonance (NMR) metabolomics (Nightingale Health Ltd). The method allows the quantification of 250 metabolic markers, including standard clinical lipids (39 of which are certified for clinical use in the European Union), along with lipoprotein subclasses, fatty acid profiles, and various low-molecular-weight metabolites such as amino acids, ketone bodies, and metabolites related to gluconeogenesis, all measured in molar concentration units. However, a key limitation of NMR is its relatively low sensitivity (ranging from millimoles to micromoles per liter), making it approximately 10 to 100 times less sensitive than mass spectrometry (MS).^24^ In addition, NMR quantifies total SMs, whereas MS can differentiate between multiple SM species. Detailed descriptions of the NMR metabolomics platform and its applications have been reported previously.^24^, ^25^, ^26^, ^27^, ^28^

#### Covariates

The following covariates were included in the analysis: waist circumference, office systolic blood pressure, body mass index, glycated hemoglobin, and plasma lipid profile.^29^ History of cardiovascular disease (yes or no), smoking status (never, former, current), and alcohol intake (none, low, or high) were assessed by questionnaire.^22^ The use of lipid-modifying agents (yes or no) and hypertension medications (yes or no) was assessed and recorded during a medication interview.^22^ The detailed methodology for the measurement of these covariates was described previously.^22^

### Statistical Analyses

#### SM Levels Across Study Groups

Sphingomyelin levels were examined across 3 study groups: NGM, individuals with prediabetes, and individuals with T2DM. Statistical comparisons were conducted using 1-way analysis of variance tests.

#### Lipid-Modifying Agents and SM Levels

Subgroup analyses were performed based on lipid-modifying agent users, that is, individuals who were using lipid-modifying agents and those who were not. Statistical comparisons were conducted using 1-way and 2-way analysis of variance tests.

#### Linear Regression Analysis

We used linear regression analysis to examine the links between plasma total SM (determinant) concentration with 3 outcome measures as follows: (1) corneal nerve measures; (2) retinal layer(s) thickness; and (3) mean retinal sensitivity independently. We standardized (std.) determinants and outcomes of a continuous nature (i.e., expressed as z-score) and entered categorical variables such as gender and GMS into models as dummy variables. Outcome measures were analyzed separately for right eye and left eye, and a combined analysis was also performed using the mean of both eyes. We included all individuals for whom data were available, for SM, outcome measures, and potential confounders required for the fully adjusted model. We used several sets of adjustments, as shown in Table 1.

**Table 1:** Regression Models

Regression Model	Determinant + Confounders
Model 1	Crude (std. SM)
Model 2	Model 1 + age, sex, educational status (middle, high), glucose metabolism status (prediabetes vs. NGM, T2DM vs. NGM), total cholesterol, total phosphoglycerides, phosphatidylcholine and other cholines, and lipid medications (yes or no)
Model 3 (Fully adjusted model)	Model 2 + waist circumference, office systolic blood pressure, smoking status (former, current), alcohol consumption status (low, high), CVD (yes or no), and hypertension medications (yes or no)

The table presents the list of covariates included in the regression models. CVD = cardiovascular disease; NGM = normal glucose metabolism; SM = sphingomyelin; std. = standardized; T2DM = type 2 diabetes mellitus.

All links were expressed as std. regression coefficients (β) with 95% confidence intervals. All analyses were performed with Statistical Package for Social Sciences version 28.0 (IBM SPSS, IBM Corp). For all analyses, a *P* value <0.05 was considered statistically significant.

#### Additional Analysis

Interaction analysis was conducted for the interaction terms as follows: prediabetes × SM (determinant), T2DM × SM (determinant), and sex × SM (determinant). For each potential confounder included in the analysis, an additional interaction term (sex × potential confounder) was incorporated into model 3. A *P* value for interaction (*P*-interaction) <0.05 was considered statistically significant.

In addition, we performed an additional analysis to examine the relationship between mean retinal sensitivity and the duration of diabetes. Duration of diabetes and its link with mean retinal sensitivity was assessed using linear regression analysis, adjusting for potential confounders, such as age, sex, education status (middle, high), total cholesterol, total phosphoglycerates, phosphatidylcholine and other cholines, lipid medications (yes or no), waist circumference, office systolic blood pressure, smoking status (former, current), alcohol consumption status (low, high), cardiovascular disease (yes or no), and hypertension medications (yes or no).

## Results

### Study Population and Baseline Characteristics

**Figure 1:**
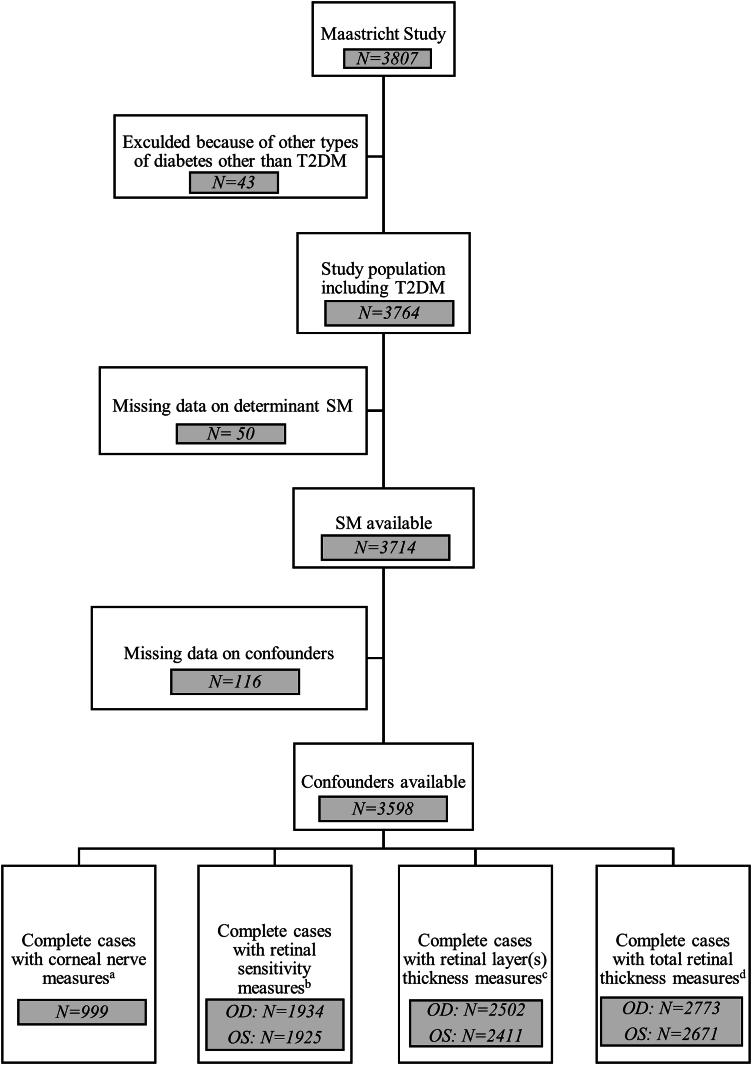
Flow diagram for selection and inclusion of *population. The number of participants excluded per outcome due to poor quality or missing images is denoted by a,b,c,d.*^*a*^*N = 2599;*^*b*^*OD: N = 1664; OS: N = 1673;*^*c*^*OD: N = 1096; OS: N = 1187;*^d^OD: N = 825; OS: N = 927. N = number of participants; OD = right eye; OS = left eye; SM = sphingomyelin; T2DM = type 2 diabetes mellitus.

After applying all exclusion criteria (Fig 1), the population for this study was composed of 2050 participants with NGM and 541 with prediabetes and 1007 participants with T2DM. Their baseline characteristics are shown in Table 2. Additional baseline characteristics of the included study population are shown in Table S3 (available at www.ophthalmologyscience.org.).

**Table 2:** Baseline Characteristics of the Included Study Participants

	NGM	Prediabetes	T2DM	*P* Value
N	2050	541	1007	
Age at visit 1 (yrs)	58.1 ± 8.3	61.6 ± 7.8	62.7 ± 7.7	<0.001∗
Sex (male)	889 (43.36%)	291 (53.79%)	678 (67.32%)	<0.001∗
BMI (kg/m^2^)†	25.5 ± 3.6	27.7 ± 4.2	29.9 ± 5.0	<0.001∗
HbA1c (%)†	5.4 ± 0.3	5.7 ± 0.4	6.9 ± 1.1	0.000∗
Serum total cholesterol (mmol/l)	5.6 ± 1.0	5.4 ± 1.2	4.5 ± 1.0	<0.001∗
SMs (mmol/l)	0.48 ± 0.08	0.45 ± 0.08	0.40 ± 0.07	<0.001∗

Data are presented as means ± standard deviation or N (%). HbA1C: NGM N = 2044, prediabetes N = 538, T2DM N = 1004. BMI: NGM N = 2049, prediabetes N = 541, T2DM N = 1007. The statistical analyses were performed using a 1-way analysis of variance (∗*P* < 0.05) for continuous variables and chi-square test (∗*P* < 0.05) for categorical variables. BMI = body mass index; HbA1c = glycated hemoglobin; N = number of participants; NGM = normal glucose metabolism; SM = sphingomyelin; T2DM = type 2 diabetes mellitus.

† Missing population.

Individuals in the prediabetes and T2DM groups had significantly lower levels of SM than those with NGM (Table 2). Additionally, individuals in the prediabetes and T2DM groups had significantly lower levels of corneal nerve measures, total retinal thickness, and mean retinal sensitivity compared with those with NGM (Table 4).

**Table 4:** Ophthalmic Measurements of Study Participants Included in the Regression Analysis

	NGM	Prediabetes	T2DM	*P* Value
N	586	144	269	
CNFL in mm/mm^2^	14.4 ± 4.4	13.3 ± 4.3	12.9 ± 4.5	<0.001∗
CNFD in number of corneal nerve fibers/mm^2^	78.4 ± 24.0	71.9 ± 25.1	71.6 ± 24.4	<0.001∗
CNBD in number of corneal nerve branches/mm^2^	69.5 ± 38.5	59.6 ± 34.8	60.2 ± 36.9	<0.001∗
N	1606	419	748	
Total retinal thickness in μm (OD)	333.7 ± 16.74	331.7 ± 22.15	328.7 ± 20.10	<0.001∗
N	1562	396	713	
Total retinal thickness in μm (OS)	333.2 ± 16.66	330.9 ± 19.90	328.7 ± 19.44	<0.001∗
N	1140	293	501	
Mean retinal sensitivity in dB (OD)	28.2 ± 1.56	28.0 ± 1.59	27.6 ± 1.91	<0.001∗
N	1147	287	491	
Mean retinal sensitivity in dB (OS)	28.1 ± 1.51	28.0 ± 1.43	27.6 ± 1.85	<0.001∗

Data are presented as means ± standard deviation, chi-square test (∗*P* < 0.05). CNBD = corneal nerve branch density; CNFD = corneal nerve fiber density; CNFL = corneal nerve fiber length; dB = decibel; N = number of participants; NGM = normal glucose metabolism; OD = right eye; OS = left eye; T2DM = type 2 diabetes mellitus.

### Links of DRN Measures with Plasma SM Levels

Although SM was not significantly associated with corneal nerve measures or retinal layer(s) thickness and SM after correction for confounding factors, there was significant positive links with mean retinal sensitivity (Table 5). Additionally, we conducted a combined analysis using the average of right eye and left eye measurements. The results were consistent with those from the eye-specific analyses and remained unchanged (data not shown).

**Table 5:** Link between SM and Indicators of Retinal Neurodegeneration

Retinal Neurodegenerative Indicators		Model 1 (β, 95%CI)	Model 2 (β, 95%CI)	Model 3 (β, 95%CI)
**Corneal nerve measures**				
CNFL	OS	0.038 (−0.023 to 0.099)	0.017 (−0.097 to 0.130)	0.011 (−0.106 to 0.127)
CNFD	OS	0.027 (−0.035 to 0.088)	0.033 (−0.081 to 0.148)	0.030 (−0.088 to 0.147)
CNBD	OS	0.014 (−0.047 to 0.076)	0.010 (−0.105 to 0.125)	0.010 (−0.108 to 0.128)
**Retinal layer(s) thickness**				
RNFL	OD	−0.006 (−0.044 to 0.033)	0.016 (−0.056 to 0.087)	0.021 (−0.052 to 0.093)
	OS	−0.001 (−0.041 to 0.039)	0.005 (−0.067 to 0.076)	0.003 (−0.070 to 0.076)
GCL	OD	**0.055 (0.016–0.094)**	0.018 (−0.05 to 0.086)	0.002 (−0.067 to 0.072)
	OS	**0.051 (0.012–0.091)**	0.010 (−0.059 to 0.079)	−0.004 (−0.075 to 0.067)
IPL	OD	**0.051 (0.012–0.090)**	0.037 (−0.032 to 0.105)	0.024 (−0.046 to 0.093)
	OS	**0.043 (0.003–0.082)**	0.023 (−0.046 to 0.092)	0.009 (−0.062 to 0.080)
INL	OD	**−0.057 (−0.096 to −0.019)**	−0.028 (−0.097 to 0.041)	−0.017 (−0.087 to 0.053)
	OS	**−0.081 (−0.121 to −0.042)**	−0.057 (−0.127 to 0.012)	−0.042 (−0.113 to 0.029)
OPL	OD	0.005 (−0.034 to 0.043)	−0.008 (−0.079 to 0.063)	−0.007 (−0.080 to 0.065)
	OS	−0.020 (−0.059 to 0.020)	0.016 (−0.056 to 0.088)	0.032 (−0.041 to 0.105)
ONL	OD	0.015 (−0.023 to 0.054)	0.025 (−0.045 to 0.096)	0.024 (−0.048 to 0.097)
	OS	0.000 (−0.039 to 0.040)	0.000 (−0.071 to 0.071)	−0.012 (−0.085 to 0.061)
PR1	OD	**0.062 (0.023–0.100)**	−0.047 (−0.115 to 0.021)	−0.058 (−0.127 to 0.011)
	OS	**0.071 (0.031–0.110)**	−0.018 (−0.085 to 0.050)	−0.031 (−0.100 to 0.038)
PR2	OD	**−0.056 (−0.094 to −0.017)**	−0.023 (−0.092 to 0.046)	−0.008 (−0.079 to 0.062)
	OS	**−0.083 (−0.122 to −0.043)**	−0.038 (−0.107 to 0.032)	−0.020 (−0.090 to 0.051)
Total retinal thickness	OD	0.016 (−0.021 to 0.053)	−0.003 (−0.068 to 0.063)	−0.010 (−0.077 to 0.057)
	OS	0.008 (−0.029 to 0.046)	−0.008 (−0.074 to 0.058)	−0.018 (−0.086 to 0.050)
**Mean retinal sensitivity**				
Mean retinal sensitivity	OD	**0.078 (0.034–0.122)**	**0.085 (0.011–0.160)**	**0.088 (0.012–0.164)**
	OS	**0.084 (0.040–0.129)**	**0.094 (0.017–0.171)**	**0.111 (0.033–0.189)**

Data are presented as standardized coefficients (β) and (95% CI); Bold denotes *P*  <  0.05. CI = confidence interval; CNBD = corneal nerve branch density; CNFD = corneal nerve fiber density; CNFL = corneal nerve fiber length; GCL = ganglion cell layer; IPL = inner plexiform layer; INL = inner nuclear layer; OD = right eye; ONL = outer nuclear layer; OPL = outer plexiform layer; OS = left eye; PR1 = photoreceptor 1; PR2 = photoreceptor 2; RNFL = retinal nerve fiber layer; SM = sphingomyelin.

### Additional Analysis

Given that the link between SM and mean retinal sensitivity was significant in model 3, we performed interaction analyses with GMS and sex by SM, as well as sex by potential confounders in the study of links between the std. SM and the std. retinal sensitivity. None of the interaction terms were statistically significant (*P* > 0.05) (Tables S6 and S7, available at www.ophthalmologyscience.org.). In the additional analysis, we found a significant negative association between the duration of diabetes and mean retinal sensitivity. This indicates that longer diabetes duration is associated with reduced retinal sensitivity, independent of other confounders (data not shown).

## Discussion

In the present study, we examined the links between levels of total SM in plasma and indicators for DRN across 3 groups, that is, NGM, prediabetes, and T2DM individuals. Our key finding is the novel link between lower plasma total SM levels and worse retinal function, as indicated by reduced mean retinal sensitivity.

Regarding a link between glycemic status and SM, we observed significantly lower levels of total plasma SM in individuals with prediabetes and T2DM compared with individuals with NGM. Findings from the present study are in line with those from another study that measured SM levels using MS, a more commonly used method for assessing SM levels. Our results, despite employing a different methodology, show similar trends, reinforcing the robustness and reliability of the NMR methodology. The total circulating SM was low in individuals with prediabetes and diabetes.^30^^,^^31^ Specifically, Xu et al^31^ showed that plasma SM species (d18:1/14:0; d18:2/16:0; d18:1/16:0) were significantly lower in prediabetes and diabetes than healthy controls, even when separately analyzed for those not on lipid-modifying medications. Likewise, we observed significantly lower total plasma SM in the T2DM group after stratification for the use of lipid-modifying medications (Table S8, available at www.ophthalmologyscience.org).

One study by Sui et al,^16^ however, found a different pattern of SM levels according to glycemic status. Levels were lower in individuals with prediabetes compared with healthy controls, but increased in the diabetes group compared with the prediabetes group. Moreover, Sokołowska et al^32^ observed a significant increase in the serum concentrations of specific SM species SM–C18:0, SM–C16:1, and SM–C18:3 in T2DM compared with nondiabetic controls. This inversed link might be explained by differential regulation on individual SM species levels.

Additionally, few studies have also investigated SM levels in ocular samples. A significant decrease in very long-chain SM species was noticed in primary human retinal endothelial cells,^33^ whereas long-chain SM species were increased in postmortem T2DM vitreous samples.^34^ Priyadarsini et al^15^ found total SM levels to be significantly decreased in corneas of individuals with T2DM. However, it is difficult to crystallize a clear T2DM-associated ocular SM pattern from these studies due to differences in the sample type, study population, and detection method.

Overall, local SM levels in the eye may be distinct from circulating levels and highly relevant for the pathophysiology of DRN. Nevertheless, evidence from previous studies points toward alterations in ocular SM metabolism in T2DM. This may be highly relevant for ocular diabetic complications, specifically the pathophysiology of DRN.

In the present study, we did not find a statistically significant link between corneal nerve measures and plasma SM levels, possibly due to the distinctive impact of SM levels on tissue and systemic levels. Although we also found no links between SM and retinal layer thickness after adjusting for confounders, we identified a significant positive link between total plasma SM with mean retinal sensitivity. This suggests a potentially supportive role of SM in maintaining neuroretinal function. This functional relationship aligns with previous findings from a longitudinal study,^14^ which reported that a specific plasma SM species (d18:2/24:2) was significantly associated with a reduced risk of diabetic retinopathy. Our findings further demonstrated a systemic reduction in circulating SM levels in individuals with diabetes compared with healthy controls, concomitant with diminished retinal sensitivity (Table 4). Such reductions in retinal sensitivity are indicative of neuronal dysfunction, potentially preceding neuronal apoptosis in the diabetic retina. Additionally, the duration of diabetes showed a negative association with retinal sensitivity (data not shown), although this was not the primary focus of the analysis.

The absence of an association between total plasma SM and retinal thickness supports the idea that functional impairments, reflected by reduced mean retinal sensitivity, may occur earlier than structural changes. This reinforces the notion that functional metrics may be more sensitive indicators of early retinal neurodegeneration. This supports the utility of SM as a biomarker of functional retinal health rather than structural loss, potentially enabling earlier detection of retinal neurodegeneration before irreversible thinning occurs.

To assess the robustness of the observed association between SM and retinal sensitivity, we conducted interaction analyses to examine the potentially modifying effects of sex and GMS. None of the interaction terms—including SM × sex, SM × GMS, or sex × potential confounders—were statistically significant (*P* > 0.05). This suggests that the relationship between SM and retinal sensitivity was consistent across these subgroups. Therefore, despite the lower proportion of males in the control group than the T2DM group, the lack of significant interactions supports the reliability of the association and reduces concerns about sex-related bias. The major strength of this study was the inclusion of a large sample size, which enabled it to account and adjust for a wide range of pertinent clinical factors. However, certain limitations should be acknowledged. Sphingomyelin measurements were taken at a single time point, preventing correlation with disease progression. Furthermore, the study lacked information on different SM species, hampering comparability with other studies that found links on SM species level. It is important to note that SM measurements in the present study were performed using NMR spectroscopy, which has lower sensitivity compared with MS. Despite this limitation, the biochemical parameters obtained through our NMR-based methodology exhibit trends that are consistent with those measured using traditional methods, underscoring the validity of our findings. Finally, follow-up experimental research is needed to reveal whether there is a causal link between reduced circulating SM and loss of retinal sensitivity, for example, due to SM-induced synaptic dysfunction.

### Conclusions

Findings of this study support the idea that low levels of total SM in plasma are linked to early retinopathic changes in the diabetic retina and, therefore, bear potential as a biomarker for early detection of DRN. Future research should focus on elucidating the mechanistic underpinnings of the link between SM dynamics and retinal health in DM individuals, with longitudinal studies being paramount to validate the translational potential of these findings. If a causal link between lower total SM and retinal pathophysiology is established, sphingolipid modulation may become a strategy for the prevention or treatment of retinal damage in individuals with diabetes.
