# Supplementary material for: Plasma Sphingomyelins as Biomarkers for Diabetic Retinal Neurodegeneration: The Maastricht Study
Source: Ophthalmol Sci. 2025 Jun 27;5(6):100870. doi: 10.1016/j.xops.2025.100870 (PMC12340381; doi:10.1016/j.xops.2025.100870)
Supplement: Supplementary Material [file mmc1.pdf]

Supplementary Materials

Supplementary Table S1. Additional general study population characteristics according to GMS

Characteristics	NGM (*N*=2050)	Pre-diabetes (*N*=541)	T2DM(*N*=1007)
Clinical Characteristics			
Fasting plasma glucose (mmol/l)#	5.2 ± 0.4	5.9 ± 0.6	7.9 ± 2.0
HbA1c (mmol/mol)#	35.9 ± 3.8	38.7 ± 4.5	52.0 ± 11.7
Serum HDL cholesterol (mmol/l)	1.7 ± 0.5	1.5 ± 0.4	1.3 ± 0.4
Serum LDL cholesterol (mmol/l)	3.4 ± 0.9	3.2 ± 1.0	2.4 ± 0.9
Serum triglycerides (mmol/l)	1.2 ± 0.6	1.6 ± 1.0	1.8 ± 1.0
Waist circumference (cm)	90.6 ± 11.1	97.8 ± 12.3	105.8 ± 13.4
Systolic Blood Pressure (mmHg)	130.8 ± 17.3	137.1 ± 16.9	141.9 ± 17.8
Diastolic Blood Pressure (mmHg)	75.2 ± 9.9	77.7 ± 9.5	77.0 ± 9.7
Total phosphoglycerides (mmol/l)	2.0 ± 0.3	2.0 ± 0.4	1.8 ± 0.3
Phosphatidylcholine and other cholines (mmol/l)	2.1 ± 0.3	2.0 ± 0.3	1.8 ± 0.3
Demographics			
Educational Level			
Low	555 (27.1%)	194 (35.9%)	470 (46.7%)
Medium	562 (27.4%)	162 (29.9%)	279 (27.7%)
High	933 (45.5%)	185 (34.2%)	258 (25.6%)
Lifestyle			
Smoking Status			
Never	806 (39.3%)	157 (29.0%)	282 (28.0%)
Former	987 (48.1%)	313 (57.9%)	564 (56.0%)
Current	257 (12.5%)	71 (13.1%)	161 (16.0%)
Alcohol Use			
None	283 (13.8%)	89 (16.5%)	306 (30.4%)
Low	1200 (58.5%)	285 (52.7%)	512 (50.8%)
High	567 (27.7%)	167 (30.9%)	189 (18.8%)

History of Systematic Diseases			
Hypertension			
Yes Vs No	857 (41.8%)	351 (64.9%)	843 (83.7%)
CVD			
Yes Vs No	255 (12.4%)	80 (14.8%)	282 (28.0%)
Medication			
Glucose Lowering Medication			
Yes Vs No	0 (0.0%)	0 (0.0%)	791 (78.5%)
Blood Pressure Lowering Medication			
Yes Vs No	462 (22.5%)	248 (45.8%)	725 (72.0%)
Glaucoma Medication			
Yes Vs No	16 (0.8%)	10 (1.8%)	22 (2.2%)
Other Ophthalmic Conditions			
High Intraocular Pressure			
Yes Vs No	51 (2.5%)	26 (4.8%)	51 (5.1%)
Missing	219 (10.7%)	57 (10.5%)	145 (14.4%)
Glaucoma			
Yes Vs No	65 (3.2%)	33 (6.1%)	67 (6.7%)
Missing	219 (10.7%)	57 (10.5%)	145 (14.4%)
Cataract			
Yes Vs No	119 (5.8%)	49 (9.1%)	133 (13.2%)
Missing	228 (11.1%)	76 (14.0%)	193 (19.2%)
Corneal Diseases			
Yes Vs No	30 (1.5%)	5 (0.9%)	21 (2.1%)
Missing	228 (11.1%)	79 (14.6%)	197 (19.6%)
Uveitis			
Yes Vs No	62 (3.0%)	18 (3.3%)	25 (2.5%)
Missing	234 (11.4%)	74 (13.7%)	198 (19.7%)
Ocular Inflammation			
Yes Vs No	88 (4.3%)	15 (2.8%)	33 (3.3%)
Missing	231 (11.3%)	76 (14.0%)	198 (19.7%)

Data are presented as mean± SD or *N (%)*

# Missing population

Fasting plasma glucose(mmol/l) : NGM N=2049, Pre-diabetes N=541, T2DM N= 1006

HbA1C : NGM N=2044, Pre-diabetes N=538, T2DM N= 1004

Effect of Lipid Modifying Agents on SM

Statistical analysis was done by one-way ANOVA separately for the groups with and without lipid-modifying medication. Table 4denotes total number of study participants distributed across two

groups i.e. those who are taking lipid modifying agents and those who are not taking lipid modifying agents. SM levels were significantly lower in T2DM compared to those with pre-diabetes and normal health conditions, irrespective of their use of lipid-modifying agents. Results from two-way ANOVA showed that both lipid modifying medication and the study groups independently influence SM levels, without interaction.

Supplemental Table S2. Plasma SM concentrations stratified by use of lipid-modifying agents and glucose metabolism status.

Lipid-modifying medication	Glycaemic status	*N* (%)	Mean SM (mmol/L)	P Value
No (*N*=2304; 64%)	NGM	1698 (74%)	0.49	<0.001*
	Pre-diabetes	347 (15%)	0.47	
	T2DM	259 (11%)	0.44	
Yes (*N*=1294; 36%)	NGM	352 (27%)	0.43	<0.001*
	Pre-diabetes	194 (15%)	0.41	
	T2DM	748 (58%)	0.38	

*The statistical analyses were performed using a one-way analysis of variance (ANOVA) (*p < 0.05)*

Supplemental Table S3. P-values for the interaction term of sex with determinant (SM) under the study in the associations of Z-score for SM with Z-score for retinal sensitivity.

Interaction Terms	Retinal Sensitivity OD (*N*=1934)	Retinal Sensitivity OS (*N*=1925)
Sex × SM	0.8	0.3

P-values represent the *P*-values for the interaction terms of sex with determinant under the study (e.g., sex × SM) in the associations of SM with retinal sensitivity. We have included interaction terms in model 3 (fully adjusted model). In addition, we added an interaction term for confounders as follows: sex*D_middle_ed, sex*D_high_ed, sex*OSBP, sex * med_LP, sex*D_former_smoke, sex*D_current_smoke, sex*D_low_alc, sex*D_high_alc., sex*Tot_chol., sex*PC_metab, sex*TotPG_metab, sex*N_CVD, sex*prediabetic vs normal glucose metabolism, sex* diabetic vs normal glucose metabolism.

Supplemental Table S4. P-values for the interaction term of GMS with determinant (SM) under the study in the associations of Z-score for SM with Z-score for retinal sensitivity

Interaction Terms	Retinal Sensitivity OD (*N*=1934)	Retinal Sensitivit y OS (*N*=1925)
Prediabetes × SM	0.6	0.1
T2DM × SM	0.8	0.2

p-values represent the *p*-values for the interaction terms of glucose metabolism status (prediabetes and type 2 diabetes versus normal glucose metabolism) with determinant under the study (e.g., prediabetes vs normal glucose metabolism × SM or type 2 diabetes vs normal glucose metabolism × SM) in the associations of retinal sensitivity. We have included interaction terms in model 3 (fully adjusted model).
